# The intersection between Descriptivism and Meliorism in reasoning research: further proposals in support of ‘soft normativism’

**DOI:** 10.3389/fpsyg.2014.01269

**Published:** 2014-11-05

**Authors:** Edward J. N. Stupple, Linden J. Ball

**Affiliations:** ^1^Division of Psychology, University of DerbyDerby, UK; ^2^School of Psychology, University of Central LancashirePreston, UK

**Keywords:** rationality paradox, normativism, radical relativism, descriptivism, soft normativism, reflective equilibrium, individual differences, reasoning

## Abstract

The rationality paradox centers on the observation that people are highly intelligent, yet show evidence of errors and biases in their thinking when measured against normative standards. Elqayam and Evans’ (2011) reject normative standards in the psychological study of thinking, reasoning and deciding in favor of a ‘value-free’ descriptive approach to studying high-level cognition. In reviewing Elqayam and Evans’ (2011) position, we defend an alternative to descriptivism in the form of ‘soft normativism,’ which allows for normative evaluations alongside the pursuit of descriptive research goals. We propose that normative theories have considerable value provided that researchers: (1) are alert to the philosophical quagmire of strong relativism; (2) are mindful of the biases that can arise from utilizing normative benchmarks; and (3) engage in a focused analysis of the processing approach adopted by individual reasoners. We address the controversial ‘is–ought’ inference in this context and appeal to a ‘bridging solution’ to this contested inference that is based on the concept of ‘informal reflective equilibrium.’ Furthermore, we draw on Elqayam and Evans’ (2011) recognition of a role for normative benchmarks in research programs that are devised to enhance reasoning performance and we argue that such Meliorist research programs have a valuable reciprocal relationship with descriptivist accounts of reasoning. In sum, we believe that descriptions of reasoning processes are fundamentally enriched by evaluations of reasoning quality, and argue that if such standards are discarded altogether then our explanations and descriptions of reasoning processes are severely undermined.

## INTRODUCTION

The rationality paradox (e.g., Evans and Over, 1996) centers on the observation that people are demonstrably highly intelligent, yet simultaneously show evidence of numerous errors and biases in their thinking, reasoning and deciding when measured against normative standards associated with formal, logical systems or probability theory. This rationality paradox has emerged from a paradigm that sets descriptions of what human thinking ‘is’ against prescriptions of what human thinking ‘ought’ to be. This paradigm is based around what Elqayam and Evans (2011) describe as ‘prescriptive normativism,’ and can be traced back to pioneering research on systematic errors in reasoning by Wason (1966) and Tversky and Kahneman (1974). The paradigm is also central to the more recent program of individual differences research by Stanovich and West (2000) and Stanovich et al. (2010) that plays squarely into a Meliorist agenda, which views people’s reasoning as being amenable to improvement through training and education. Elqayam and Evans (2011), however, have presented a powerful critique of ‘normativism’ in reasoning research, whether of the prescriptive variety favored by Meliorists or of the ‘empirical’ variety, favored by Panglossian theorists (e.g., Oaksford and Chater, 2007), who propose that human reasoning is *a priori* rational, having been forged by adaptive evolutionary forces that have patterned fitness-relevant characteristics that enable effective goal attainment.

Elqayam and Evans’ (2011) critique argues that both prescriptive and empirical normativism invite researchers to make a logically contested ‘is–ought’ inference. In the case of prescriptive normativism, when there are competing normative accounts then empirical ‘is’ evidence is inevitably called upon as a basis for arbitration, giving rise to a clear case of is–ought reasoning. For example, Stanovich and West (2000) have proposed that the reasoning of the most cognitively able respondents can arbitrate between opposing normative accounts (cf. Stich and Nisbett, 1980). In the case of empirical normativism, the is–ought inference arises by virtue of the Panglossian view that ‘average’ or ‘modal’ responses that occur on reasoning tasks are an index of normative reasoning (e.g., Cohen, 1981; cf. Oaksford and Chater’s, 2007, rational analysis approach). Elqayam and Evans (2011) contend that normativism in both of these guises should be strictly avoided given that the dubious is–ought inference that it invokes fosters misunderstandings and obstructs sound theorizing. They instead advocate a descriptivist analysis of reasoning as the only viable way forward for the study of high-level cognition. Elqayam and Evans (2011) further suggest that there is an acceptable role for ‘formal systems’ in theory development when such formal systems are applied in a non-evaluative manner. In this respect they propose that logical systems or probability theory can be useful in providing a ‘computational-level’ analysis (Marr, 1982) or a ‘competence’ theory (Chomsky, 1965) in terms of offering structural descriptions of people’s abstract knowledge that are nevertheless ‘value-free.’ It is also noteworthy that Elqayam and Evans (2011) propose that normative approaches *can* be useful in one very restricted sense, that is, when the researcher has an applied objective “to improve thinking (rather than understand it)” (p. 242), since it is then necessary to have criteria that can distinguish good thinking from bad thinking.

In this paper we set out to defend an approach that can be seen as a middle ground between a descriptivist perspective and a stance that is based on prescriptive normativism. The approach that we advocate has been dubbed ‘soft normativism’ by Evans and Elqayam (2011), and is a position that sees a role for normative evaluation in reasoning research alongside the pursuit of descriptive research goals. Our argument (cf. Stupple and Ball, 2011) proposes that normative theories have considerable value for formulating and testing hypotheses, provided that researchers: (1) remain alert to the philosophical quagmire of more radical forms of relativism (e.g., Stich, 1990; Elqayam, 2012); (2) are mindful of the biases and pitfalls that can arise from drawing on normative accounts of reasoning (see Elqayam and Evans, 2011); and (3) ensure that they engage in a focused analysis of the processing approach adopted by *individual* reasoners when confronted with reasoning tasks (cf. Stanovich and West, 2000).

In this paper we will examine the position of soft normativism in the context of dual-process theories of reasoning, individual differences in reasoning and attempts to ameliorate reasoning ‘defects.’ Our conclusion is that descriptions of reasoning processes are fundamentally enriched by evaluations about the quality of that reasoning. As such, soft normativism is, we suggest, a reasonable pragmatic position to take when both judging reasoning and when formulating theoretical accounts. In developing our argument we also address how soft normativism can circumvent the contested is–ought inference by means of a well-recognized bridging solution (see Evans and Elqayam, 2011) that is based on the concept of reflective equilibrium (Goodman, 1965). We extend the notion of reflective equilibrium to capture the way in which the reasoning behavior of naïve individuals changes when they are provided with extensive opportunities to practice their reasoning.

We additionally propose that the distinction between the applied science of ‘improving thinking’ versus the pure science of ‘understanding thinking’ it not a clear-cut dichotomy of the kind that Evans and Elqayam might like to envisage. Because of this overlap our own position sits at the intersection between Meliorist and descriptivist research agendas. On the one hand we believe that to inform efforts to enhance reasoning and argumentation we must have a good understanding of underlying reasoning processes, since this will aid our explanation of why some individuals are better at making arguments or drawing inferences than others. On the other hand, the converse relationship is also important, since understanding the way in which Meliorist approaches are effective in enhancing reasoning can supplement our theoretical understanding of underlying reasoning processes. In other words, studying the improvements that can arise in thinking, reasoning or judgment through training or educational interventions allows researchers to draw important comparisons between what people can achieve as a result of such external guidance (coupled with their own reflective process) and what people can achieve through a spontaneous process. This contrast between a ‘sophisticated’ versus ‘naïve’ reasoning process is psychologically interesting and arises directly from Meliorist researchers’ attempts to align reasoning with external, normative benchmarks.

## STRONG NORMATIVISM AND RADICAL RELATIVISM

Extreme views at either end of the normativism–descriptivism spectrum are beset with problems. A strong Panglossian normativist such as Cohen (1981) must be able to demonstrate that all errors of reasoning can be explained away through lapses of attention, misunderstandings of instructions or (ecologically invalid) cognitive illusions. Stanovich and West (2000), however, have convincingly demonstrated that if errors are predominantly the result of lapses of attention then such errors within tasks should be uncorrelated since these lapses will be randomly distributed, and likewise performance across tasks should also be uncorrelated for the same reason. A wealth of evidence, however, has shown this not to be the case, with systematic errors and biases being demonstrated within tasks and with clear correlations arising between various reasoning and judgment tasks. The correlations in performance across tasks are not without exceptions^1^, but do nevertheless provide evidence that is problematic for the view that the so-called ‘normative–descriptive gap’ (Stanovich and West, 2000) can be explained within the framework of pure normativism.

Moreover, Stich (1990) presents further challenges for adherents of strong normative positions with his concept of ‘cognitive pluralism’: that there is more than one good way to reason. Stich illustrates this view with comparisons to alternative cultures that may not share Western ideals about particular normative systems, and he further extends this position (via Goldman, 1986) to ask whether a normative system must hold in all possible worlds (or at least ‘normal worlds’) if it is truly universal. If we concur with Stich’s argument then we have stepped onto the slippery slope to radical relativism and have accepted that there is no universal benchmark to judge inferences (or to make judgments about judgments) that can apply to all contexts. If we continue all the way to the bottom of this slippery slope then we reach the anarchic conclusion that all inferences and choices are equal. Buckwalter and Stich (2011) note, however, that the concept of cognitive pluralism made little headway initially, and they suggest that this was because there was no compelling evidence that it is ‘psychologically’ possible for people to have significantly different reasoning competences. They concur, however, that Stanovich’s (1999) research program on individual differences in reasoning goes a fair way toward demonstrating that there are indeed a range of such competences. Although this does not indicate that all possible inferences are justifiable, it nevertheless indicates that a degree of relativism may be undeniable when it comes to describing reasoning competence. The concept of cognitive pluralism advanced by Stich not only poses problems for using normative benchmarks as standards to judge thinking and reasoning, but also has the potential to be challenging for computational-level or competence-based descriptive accounts of thinking and reasoning by virtue of the need for an explanation of why such varied competences arise.

We nonetheless defend a moderate relativism by noting that the ‘slippery slope’ argument is a well-known fallacy such that we can make progress by recognizing the limitations of accounts that assume normative benchmarks and cognitive universality, while also acknowledging some of the important issues that normative views raise in defense of human rationality. Indeed, Samuels and Stich (2004) have likewise argued for a ‘middle way’ when conceiving of human rationality in the context of dual-process theory, which they see as offering an escape from the rationality paradox. It is in a similar spirit that we argue for the application of a softer normativism when engaging in reasoning research. The soft normativism that we advocate is admittedly a few steps down the slippery slope toward relativism in that it recognizes a role for context and participant knowledge in judging the efficacy or appropriateness of an inference. Nevertheless, our proposed soft normativism still places considerable value on people’s ability to produce valid inferences in response to reasoning and decision making tasks.

The crux of our position is that while we endorse the use of normative standards as the basis for a descriptively oriented computational level of analysis (or what might also be viewed as a ‘competence theory’ of reasoning; Elqayam and Evans, 2011), and while we also acknowledge that from a descriptivist perspective there is no additional value in regarding deviations from these standards as ‘errors,’ we still believe that normative standards can benefit the applied study of reasoning provided that they are deployed sensibly. We advocate the use of normative standards – in accordance with Elqayam and Evans (2011) – where the goal of research is to enhance reasoning, argumentation or judgment so as to align it with external benchmarks of quality. Certainly a primary goal of Meliorist researchers is to increase the proportion of people who avoid bias and endorse some set of external normative standards (i.e., is the desire is to ensure that people reason as well as they are able to). This Meliorist agenda represents a substantial research program within the discipline of Cognitive Science that is either followed explicitly (e.g., Stanovich, 2011) or else implicitly (e.g., Ball, 2013b). We contend that it is simply not possible to have such a Meliorist agenda without some notion of what constitutes ‘good’ thinking. We further assert that while there can be a range of normative theories that apply to a particular reasoning or decision making domain (i.e., we accept that these standards can be controversial), they still offer us a guide as to what constitutes good reasoning or judgment. Thus, when a Meliorist researcher succeeds in enhancing thinking, this change in performance (or even the capacity to change) needs to be compatible with a computational-level explanation (i.e., the descriptivist researcher needs to be able to explain the Meliorist researcher’s findings). In this latter respect we believe that a soft normativist compromise is what is required to allow for a mutually beneficial symbiosis between Meliorist and descriptivist agendas.

## PITFALLS WHEN DRAWING ON NORMATIVE THEORIES

Theorists such as Cohen (1981) have argued that the reasoning research paradigm has not been particularly charitable to participants over the years, with a tendency to present ‘trick’ questions with minimalist instructions to naïve individuals. Judging non-normative responses as indicative of irrationality on this basis is, he proposes, difficult to justify. Evans (2007) has argued that researchers such as Cohen who attempt to defend human rationality have tended to do so by appealing to three key problems with the attribution of irrationality to reasoners: (1) the normative system problem; (2) the interpretation problem; and (3) the external validity problem (see also Evans, 1993; Evans and Over, 1996). The normative system problem is that researchers are simply applying the wrong normative standards when judging participants’ task performance, such that if the correct normative system were applied then behavior could be re-classified as rational. It is worth noting that there are many logical systems (e.g., see Garson, 2014) and that this diversity has provoked debate about which normative system is the ‘correct’ one in any particular reasoning context. Such diversity can also prompt interesting questions as to the characteristics of individuals who endorse differing benchmarks when multiple standards are available. For the Meliorist, however, it is inevitable that there will be a degree of ‘satisficing’ when selecting a normative standard against which to judge reasoning or decision making, since such standards can be debatable and can develop and change through cultural evolution as tools of rationality. As Stanovich (2011) notes: “… there is no idealized human ‘rational competence’ that has remained fixed throughout history” (p. 269).

The interpretation problem explains participants’ deviations from normative theory not in terms of the application of faulty reasoning processes but instead in terms of participants adopting alternative mental representations of problem information to that intended by researchers. The external validity problem, which is closely allied with Cohen’s (1981) argument noted above, is that the tasks that researchers select in order to demonstrate human irrationality are not at all representative of the tasks that arise in real-world contexts, which tend to be associated with normatively accurate reasoning. Evans (2007) argues that the interpretation problem and the external validity do not hold up to close scrutiny because they fail to offer ‘complete’ accounts of the discrepancy between normative benchmarks and actual behavior. We would counter, however, that neither approach needs to offer a comprehensive account of normative–descriptive discrepancies so long as each approach can offer up explanations of at least some of the relevant data. Take, for example, the interpretation problem as discussed by Evans (2007). There is a good degree of consensus in the literature (e.g., Stanovich and West, 2000) that there are individual differences in cognitive ability and thinking dispositions that influence reasoning. There is, moreover, evidence of individual differences in the *interpretation* of elements of the reasoning scenarios and vignettes that participants tackle in the laboratory (e.g., Roberts et al., 2001; Stenning and Cox, 2006). For example, if an individual fails to interpret the quantified assertion *Some A are B* as possibly meaning *All A are B*, then this invites the assumption that *Some A are not B* is also true (Newstead and Griggs, 1983)^2^. Newstead (1989) demonstrated that quantifier interpretation can indeed influence performance in some circumstances, and Roberts et al. (2001) showed that while there can be ‘errors’ based on interpretation, these vary according to the complexity of the task. Stenning and Cox (2006) further revealed that individual differences in the interpretation of quantifiers result in differing patterns of responses. In sum, it seems important to acknowledge that the interpretation problem is a very real one, even if it does not provide a complete explanation of deviations from normative benchmarks in all situations and even if explaining the findings that arise in studies is not always straightforward.

This interpretation problem in reasoning research also has implications for explaining reasoning accuracy in the context of dual-process theories that invoke a distinction between rapid, effortless and intuitive ‘Type_1_’ processes and slow, effortful and analytic ‘Type_2_’ processes (e.g., Evans and Stanovich, 2013). The predominance of naive participants in reasoning studies means that some task misinterpretation is inevitable, which confounds any inferences that researchers might want to make either about non-normative responding reflecting Type_1_ processing or about normative responding reflecting Type_2_ processing (see also Thompson, 2011). In the latter case, for example, if quantifiers in syllogistic reasoning tasks are misinterpreted then non-normative responding might still be based on effortful Type_2_ thinking. In this respect we are reminded of Smedslund’s (1990) critique of Kahneman and Tversky’s (1972) heuristics and biases paradigm, whereby he argued that we cannot decide if someone has reasoned logically unless we assume they represented the premises as the experimenter intended, and likewise we cannot judge whether someone represented the premises as intended unless we assume they reasoned logically. This circularity continues to be an issue when equating normative responses with Type_2_ processing (e.g., see Evans, 2012). For example, someone could employ a normative goal (i.e., to reason logically) and pursue this goal with great effort using Type_2_ processing, and yet still offer a non-normative response because they are unaware of the need for a ‘non-pragmatic’ interpretation of a quantifier (i.e., an interpretation that is inconsistent with everyday usage). In fact, Noveck and Reboul (2008; see also Bott and Noveck, 2004) have shown that effortful processing is required to narrow *Some* to *Some but not all*, which means that in some cases a pragmatic interpretation may require more Type_2_ processing than a normative response.

Whilst these aforementioned issues might be seen to undermine entirely any agenda that attempts to align participants’ responses with normative theories, we would argue instead that such issues simply alert researchers to the need for more cautious interpretation of reasoning data. Indeed, we would go a step further and propose that such issues can guide the careful design of experiments in the first place so that they can accommodate the way in which participants are likely to engage in pragmatic interpretations of information. An example of just such an approach comes from a study by Schmidt and Thompson (2008), who used the quantifier *At least one and possibly all* instead of *Some* within given premises and found that participants were facilitated in giving normative responses. Whilst the deductive paradigm and instantiations of it, such as the belief-bias paradigm^3^ (e.g., Evans et al., 1983; Stupple and Ball, 2008), continue to be important test-beds for dual-process accounts of reasoning, we advocate the increased utilization of pragmatically interpretable quantifiers (or else instructions regarding how quantifiers should be interpreted) in order to increase precision when deciphering apparent variations between normative benchmarks, descriptions of performance and the alignment of outputs with Type_2_ processing.

## THE IMPORTANCE OF DATA TRIANGULATION IN EVALUATING THE NORMATIVE BASIS OF REASONING

Given that pragmatic interpretations and responses can explain some (but not all) deviations from normative standards, we believe that it is increasingly important to include the triangulation of measures (e.g., response types, processing times, and confidence judgments) in any empirical studies that are examining the nature of reasoning, including its normative basis and possible dual-process components. In this respect it has been encouraging to see a burgeoning over the past decade or so in the use of ‘multi-method’ approaches in reasoning research (for good examples of such multi-method studies see Quayle and Ball, 2000; Thompson et al., 2003, 2011a,b, 2013; De Neys, 2006; Stupple and Ball, 2007, 2008; De Neys and Glumicic, 2008; Prowse Turner and Thompson, 2009; De Neys et al., 2011; Stupple et al., 2011). Particularly valuable insights into the nature and time-course of reasoning processes can be gained by examining think-aloud protocols that are acquired from participants who are tackling reasoning problems (e.g., Evans et al., 1983; Lucas and Ball, 2005; Wilkinson et al., 2010) as well as by analyzing neuroimaging data collected concurrent to reasoning performance (e.g., Goel and Dolan, 2003; Luo et al., 2013)^4^. Houdé (2007) has, in fact, recently argued that “… one of the crucial challenges for the cognitive and educational neuroscience of today is to discover the brain mechanisms that enable shifting from reasoning errors to logical thinking” (p. 82). The challenge that Houdé refers to clearly requires a major drive toward the increasing deployment of triangulating measures that attempt to understand the neural underpinnings associated with the transition that people are able to make toward normative responding through training and education. A recent example of such an approach comes from Luo et al. (2014) who demonstrated differences in activation for the left inferior frontal gyrus, left middle frontal gyrus, cerebellum, and precuneus for a group of highly belief-biased participants who had subsequently received logic training and switched to logic-based responding.

A further monitoring approach for examining the dynamic aspects of reasoning that we are particularly enthusiastic about is to deploy eye-tracking (e.g., Ball et al., 2003, 2006) to determine the moment-by-moment attentional shifts in processing that arise when participants attempt the visually presented problems that are typically used in reasoning studies (see Ball, 2013a, for a recent summary of key findings deriving from eye-tracking research in reasoning). Eye-tracking studies have, we contend, provided some of the most compelling evidence to date that Type_2_ analytic reasoning that is attuned to normative principles plays an important role in determining whether heuristically cued cards are subsequently selected or rejected in the Wason four-card selection task (see Evans and Ball, 2010). Likewise, eye-tracking studies of belief-bias effects (e.g., Ball et al., 2006) have been influential in revealing that people spend longer reasoning about ‘conflict’ syllogisms, where conclusion validity and believability are in competition (i.e., those with invalid-believable conclusions and valid-unbelievable conclusions), relative to ‘non-conflict’ syllogisms, where conclusion validity and believability concur (i.e., those with valid-believable and invalid-unbelievable conclusions). The evidence that conflict problems take longer to process than non-conflict problems is viewed by Stupple and Ball (2008) as indicating that participants are ‘sensitive’ to the fact that the logic of a conclusion and its belief status are in opposition such that extra processing effort has to be allocated to resolving the conflict. Such findings resonate with recent proposals that have been forwarded by De Neys (2012), who suggests that people’s indirect sensitivity to the normative status of presented conflict conclusions is indicative of their possession of an ‘intuitive logic’ (a Type_1_ process) that functions implicitly and in parallel to implicit heuristics (also Type_1_ processes) to signal the need for Type_2_ processing. De Neys (2014) presents some clarifications about the role of these controversial ‘gut-feelings’ in shaping the way participants respond to conflict problems and asserts that whether or not we endorse his ‘logical intuition’ proposal we can certainly question the idea that Type_1_ responses can typically be attributed to a failure in conflict detection. We are mindful, however, of the calls from Singmann et al. (2014) for the application of the most rigorous, scientific approach possible when examining such ‘extraordinary’ claims as the existence of an intuitive logic (see also Klauer and Singmann, 2013).

One important area of eye-tracking research in the reasoning domain that is currently gaining increased attention concerns the analysis of eye-movement metrics that are directly linked to people’s *comprehension* of visually presented logical statements. For example Stewart et al. (2013) deployed eye-tracking to examine how readers process “if … then” statements used to communicate conditional speech acts such as promises (which require the speaker to have perceived control over the consequent event) and tips (which do not require perceived control). Various eye-tracking measures showed that conditional promises that violated expectations regarding the presence of speaker control resulted in processing disruption, whereas conditional tips were processed equally easily regardless of whether speaker control was present or absent. Stewart et al. (2013) concluded that readers make very rapid use of pragmatic information related to perceived control in order to represent conditional speech acts as they are read. These kinds of on-line studies of ‘reasoning as we read’ (see also Haigh et al., 2013) seem likely to open up many new possibilities for advancing an understanding of reasoning processes by providing converging empirical evidence to help arbitrate between competing theoretical accounts.

Overall, we contend that without alternative, convergent measures of reasoning that extend well beyond mere response choices we have no direct gage of the nature and time-course of reasoning, such as whether the cognitive processing that participants deploy is slow and effortful or fast and intuitive. Furthermore, simply knowing that responses are consistent with normative benchmarks is clearly insufficient to claim that Type_2_ thinking is involved (e.g., Evans and Stanovich, 2013; see also Evans, 2012, for important arguments and evidence in this respect). A recent illustration of this point comes from Stupple et al. (2011), who demonstrated correlations between response times and normative responding in a belief-bias paradigm, with increased response times to invalid-believable problems being indicative of increased normatively aligned performance. Thus, those participants who exhibited longer response times where there was a conflict between belief and logic, and who identified invalid-believable conclusions as ‘possibly true’ rather than ‘necessarily true’ (which requires a more complex understanding of *Some … are not …* than the standard pragmatic interpretation), appeared to possess the requisite cognitive resources and motivation to search for counterexamples. This meant that these participants were more likely to respond normatively to belief-oriented problems in general, and not just to the invalid-believable conflict items.

Similarly, Stupple et al. (2013) investigated ‘matching bias’ in syllogistic reasoning from a dual-process perspective. Matching bias is the phenomena whereby responses are simply matched to terms mentioned in a rule or are based on the surface features of premises, in either case being based on a ‘non-logical’ process (e.g., see Evans and Lynch, 1973; Wetherick and Gilhooly, 1995). In Stupple et al.’s (2013) study the surface features of problems were manipulated so as to be either congruent with or orthogonal to the logic of the presented conclusions. Performance was then judged based on whether it aligned with the surface features of the problems or with normative responses as determined by formal logic. This experimental set-up is much like the belief-bias paradigm, where conclusion believability and validity either concur or conflict. To manipulate the surface features of problems Stupple et al. (2013) used premises and conclusions that were matched or mismatched in terms of the presence of double negated quantifiers (e.g., *No A are not C*) or in terms of the presence of standard affirmative quantifiers (e.g., *All A are C*). Using this paradigm Stupple et al. (2013) revealed some important parallels between their results and findings deriving from studies of belief-bias. One key parallel concerned the observation that ‘conflict’ problems in both paradigms show inflated response times relative to non-conflict problems (cf. Thompson et al., 2003; Ball et al., 2006; Stupple and Ball, 2008; Stupple et al., 2011), which is entirely in line with dual-process predictions and attests to the value of obtaining response-time data as a way to inform theorizing. Stupple et al.’s (2013) study also revealed that the supposedly ‘intuitively obvious’ deduction of *double negation elimination* (see Rips, 1994, pp. 112–113) was demonstrably unintuitive for a number of participants, who showed increased response times to problems involving such negations.

Perhaps of more pertinence to the present discussion are Stupple et al.’s (2013) findings from the same study that contrasted with what has previously been observed for problems within the standard belief-bias paradigm, particularly in relation to correlations between response times and normative response rates. In particular, valid non-matching ‘conflict’ problems actually revealed an association between normative responding and *faster* responses, which is distinct from what is seen in belief-bias research, where valid-unbelievable conflict items show an association between normative responding and *slower* responses (e.g., Stupple et al., 2011). To explain this discrepancy Stupple et al. (2013) proposed that motivated participants who do not possess the double elimination rule (or who have difficulty applying it) might engage in a misdirected and slow analytic process to find a matching-consistent answer (see Stupple and Waterhouse, 2009; Stupple et al., 2013), whereas participants who eliminate the double negation are confronted with little cognitive demand in identifying that the conclusion is necessarily true such that they can rapidly respond normatively. We suggest that without the reference point that normative benchmarks offer, such idiosyncrasies in individual responding may well pass unnoticed. The combination of cognitive effort, quantifier interpretation and cognitive disposition demonstrate the increasing importance of individual differences approaches in reasoning research and also illustrate the utility of having normative benchmarks as a point of comparison.

In the next section we discuss in more detail the value of adopting an individual differences perspective on reasoning strategies whilst also further examining the way in which normative reference points can benefit an understanding of reasoning data. First, however, we take a brief detour into another area of contemporary reasoning research that also exemplifies the importance of methodological triangulation, that is, research on metacognition and reasoning – or so-called ‘meta-reasoning’ (for a recent review see Ackerman and Thompson, 2014; for pioneering conceptual work see Thompson, 2009). This growing research topic is concerned with the processes that ‘regulate’ reasoning, for example, by setting goals, deciding among strategies, monitoring progress and terminating processing. The meta-reasoning framework is predicated on the assumption that people are generally motivated to attempt to provide ‘right’ answers to reasoning problems. Indeed, meta-reasoning is centrally concerned with an individual engaging in processes such as determining how much effort to apply to the problem, assessing whether a solution that they have generated is correct, and deciding whether to initiate further processing if a putative solution seems in some way inadequate (Thompson, 2009; Ackerman and Thompson, 2014). As a case in point, Ackerman and Thompson (2014) suggest that the very first decision that that a reasoner should make is that of whether to attempt a solution at all, since the individual might determine that the amount of effort they need to apply to achieve a solution is greater than the perceived benefit of solving the problem (cf. Kruglanski et al., 2012). Ackerman and Thompson (2014) suggest that such ‘Judgments of Solvability’ are likely to be based on a range of factors, including beliefs about the task at hand, prior experience of solving similar problems, as well as surface-level cues within the problem itself that might signal difficulty, such as the ease with which the problem can be mentally represented (e.g., Quayle and Ball, 2000; Stupple et al., 2013) or the perceived coherence amongst problem elements (e.g., Topolinski and Reber, 2010; Topolinski, 2014).

As can be seen in relation to Judgments of Solvability, the meta-reasoning framework presupposes that people do not have direct access to their underlying reasoning processes, but instead base their monitoring and regulation judgments on their experience with similar problems as well as on available cues associated with the problem being tackled. One particularly important cue is that of ‘fluency,’ which is the ease or speed with which a solution to a reasoning problem comes to mind (e.g., Alter and Oppenheimer, 2009; Ackerman and Zalmanov, 2012). Thus, an individual will generally view an initial response that is produced fluently as being accurate, whereas an initial response that is difficult to generate will give rise to a sense of unease in relation to its accuracy, often triggering further processing effort. Importantly, such heuristic cues to accuracy may not be valid predictors of normative correctness, leading to some striking dissociations between participants’ response confidence and normative standards of accuracy (e.g., see Shynkaruk and Thompson, 2006; Prowse Turner and Thompson, 2009; De Neys et al., 2013). Thompson (2009) and Thompson et al. (2011b, 2013) have gone beyond the basic concept of answer fluency in their theorizing to suggest that such fluency mediates a judgment that they term ‘Feeling of Rightness.’ It is this Feeling of Rightness judgment that then acts as a metacognitive trigger, either: (1) terminating processing in cases where a Type_1_ process has readily produced a rapid, intuitive answer that is attributed to be correct; or (2) switching from Type_1_ to Type_2_ processing in cases where the initial, intuitive answer is associated with a low Feeling of Rightness and is therefore attributed to be potentially incorrect (see Ackerman, 2014, for further evidence and model development regarding people’s time investment in reasoning).

In sum, recent evidence gives clear grounds for viewing meta-reasoning judgments as playing a crucial role in monitoring and regulating on-going reasoning, such that intermediate confidence or ‘rightness’ assessments determine the amount of subsequent effort that reasoners invest in a task (Ackerman, 2014). The methodology underpinning this meta-reasoning research is based on a rich triangulation of measures, including various forms of confidence judgments as well as processing times and normative response accuracy. Analyzing confidence ratings in conjunction with other measures also seems advantageous in terms of distinguishing between normatively incorrect answers that participants ‘expect’ to be correct with high probability and wild guesses (i.e., responses made with particularly low confidence), which perhaps reflect task abandonment and may therefore be of less theoretical interest.

We suggest that the evident tendency in meta-reasoning research to evaluate participants’ responses against normative benchmarks such as logic, suggests that this emerging research tradition has a strong normative orientation, which is also bolstered by the inherent assumption underpinning the approach that participants are generally striving to produce ‘right’ answers to problems. Notwithstanding our view that normative considerations have an important role to play in emerging research on meta-reasoning, we do nevertheless concur with Thompson’s (2011) argument that simply knowing that a final outcome is normative tells us virtually nothing about underlying mechanisms. At the same time, however, we believe that a combination of process-oriented analyses together with the normative assessment of outcomes provides for a maximally rich and meaningful approach to reasoning research, especially when combined with studies of the roles of learning, practice and feedback in reasoning, as discussed below. These themes tap directly into a Meliorist research agenda, where evaluations of normative correctness are crucial. In this respect we look forward to further research using measures such as Judgment of Solvability and Feeling of Rightness in the context of training reasoning through instructions, practice, and feedback. We believe that such work could inspire new insights into the monitoring and regulatory processes that lead to both normative and non-normative reasoning responses, whilst also benefiting applied research on improving reasoning (see Ackerman and Thompson, 2014, for discussion of numerous real-world domains that could be enhanced through such research, such as innovative product design and financial decision making).

## THE IMPORTANCE OF FOCUSING ON INDIVIDUAL DIFFERENCES

Since the seminal research of Gilhooly et al. (1993), Roberts (1993), and Ford (1995) there has been a snowballing of individual differences studies in reasoning research, perhaps best exemplified by the work of Stanovich and colleagues (e.g., Stanovich and West, 2000). The question of why some participants respond in accordance with normative standards more frequently than others forms an important research agenda in its own right, but the ability to account for individual differences within a particular theoretical framework is increasingly part of the debate in a range of reasoning research paradigms (e.g., Stupple et al., 2011; Trippas et al., 2013). Nickerson (2008) argues that determining which normative system is the best one in a given context is often an uninteresting issue, unless it also happens that aligning cognitive processing with the normative system in question also correlates with something that people care about. A strong supporter of a normativist agenda could argue that since Stanovich and colleagues have demonstrated correlations between tasks from the reasoning and decision making literature with things that are prized – such as SAT scores – then it is possible to believe that there is something valuable in adhering to these normative standards. If our instrumental goals are to gain a place at a prestigious university or to score well on an employer’s recruitment test of cognitive ability, then reasoning and deciding in accordance with normative benchmarks can be an instrumental goal, at least for some participants some of the time^5^.

There is, nevertheless, much debate concerning the issue of how normative standards can be derived in the first place. The concept of ‘reflective equilibrium’ is central to this debate, and was a notion that was advanced by Goodman (1965), who argued that as the rules of deduction are determined by accepted deductive practice then good deductive rules are retained and poor deductive rules that lead to poor inferences are dropped. This is a rigorous circular process that is engaged in by philosophers and logicians in developing normative standards of inference. This concept was further developed by Cohen (1981) in the context of the rationality paradox. The idea is that normative theory and descriptive evidence can justify each other by being brought into coherence such that there is an alignment between norms and behaviors. As Elqayam and Evans (2011) note, reflective equilibrium is a ‘bridging solution’ to the notorious is–ought problem since it presupposes that full coherence is entirely possible between norms and behavior inasmuch as they become mutually justificatory.

Of course, the proposal that reflective equilibrium can offer a route to deriving appropriate normative benchmarks is not without its critics, with Stich (1990), for example, emphasizing that it has the potential once again to lead down the slippery slope to radical relativism. Stich argues that the gambler’s fallacy and base rate neglect pass many people’s tests of reflective equilibrium, which indicates that the principle can be flawed as a means of justifying inferences. Stich also demonstrates that the issue cannot be solved if we impose restrictions on the people whose reflective equilibrium is considered to be sufficiently rigorous to serve as a justification, since even experts could “end up endorsing a nutty set of rules” (p. 86). There may also be cultural and interpersonal differences in assessing the justification of an inference that yield different benchmarks in different contexts. For many, Stich’s critique would appear terminal for the use of reflective equilibrium as a means of justifying universal norms for inference. Nevertheless, his critique does not entirely rule out the application of similar principles by individuals in justifying their own inferences and judgments. Indeed, it is possible that participants can engage in an informal process analogous to reflective equilibrium in establishing how they should respond to reasoning tasks.

Recent findings by Ball (2013b) advance this aforementioned concept of ‘informal reflective equilibrium’ by indicating that participants will, through repeated reasoning practice, develop their own benchmarks for accuracy. This observation seems further to support a moderate relativism that functions hand-in-glove with soft normativism. Ball (2013b), for example, demonstrated that participants who repeatedly engaged in reasoning with belief-oriented syllogisms that are known to be susceptible to a non-logical belief-bias became steadily more normatively justified in their responding over time. This trend toward increased normative responding was seen to arise even more quickly amongst those receiving feedback regarding the logical appropriateness of their decisions. Ball’s findings suggests that through mere engagement and increasing familiarity with reasoning tasks people can self-determine a strategy that can affect a logical solution. Such evidence suggests a novel perspective on reflective equilibrium that is not based so much on what the most cognitively able do or what the majority do, but which instead is based on what individuals do when provided with opportunities for practice. This type of informal or ‘naïve’ reflective equilibrium admittedly lacks the rigor of the approach advanced by Goodman (1965), but it nevertheless indicates that untrained participants can align themselves with normative benchmarks without explicitly knowing that they are doing so or receiving feedback indicating that this is the case. Not all participants succeed in such normative alignment, and it could be argued that there is an element of ‘satisficing’ entailed in this process (e.g., see Evans, 2006, 2007), whereby individual differences in cognitive ability, disposition and motivation may all play an important role.

The present claims regarding the concept of informal reflective equilibrium – as well as Ball’s (2013b) empirical evidence – seem to chime with the radical idea mentioned earlier that people may have ‘logical intuitions,’ as demonstrated, for example, by their decreased confidence when rejecting normative responses and endorsing non-normative responses (e.g., De Neys, 2012, 2014; De Neys and Bonnefon, 2013; see also Stupple et al., 2013). In Ball’s (2013b) study the steadily increasing normative responding that was observed over time by the participants who did not receive feedback might well have been shaped by a repeated sense of metacognitive dissatisfaction with proffered answers – arising from ‘logical intuitions’ – in cases where such answers contradicted normative benchmarks. An alternative view is that through repeated exposure to belief-biased problems, the Type_2_ analytic process becomes better attuned to the problem structure and participants become increasingly aware of the role of counterexample models in invalidating presented conclusions, irrespective of their belief status. Such issues warrant further investigation, but a purely descriptivist approach to reasoning research would rule out the use of logic as a normative reference point when scrutinizing participants’ responses and would, moreover, seem to render these avenues of investigation out of bounds, irrespective of their scientific merit.

If we disallow normative theories from being utilized to inform the development of research paradigms we believe that we are, in fact, introducing a new benchmark for conducting reasoning research that is potentially obstructive to progress. For example, if the use of counterexamples is useful for good argumentation (e.g., Weston, 2009) then it is not only important to encourage our students to consider counterexamples to improve their arguments, but also for us as cognitive psychologists to understand the processes whereby individuals become attuned to the need to consider counterexamples in order to reason better. More generally, by understanding the underlying cognitive processes, we can better inform methods for improving thinking, but this would be hampered if we were not able to make value judgments about the way that participants approach their task. As another example we again refer to the belief-bias study by Stupple et al. (2011) that we outlined previously, which demonstrated that the most normatively consistent reasoners with belief-oriented syllogisms were those who had inflated response times for a particular item type that required the consideration of counterintuitive counterexamples. Stupple et al.’s (2011) evidence reconciled the descriptivist ‘selective processing theory’ of Evans (2000) with a previously conflicting data-set arising from a study by Stupple and Ball (2008). In addition, Stupple et al.’s (2011) evidence was informative from a Meliorist perspective, since it highlighted elements of reasoning tasks that are particularly demanding whilst also revealing individual differences in processing that correlate with solution success.

When participants engage in reasoning experiments they are likely to assume there are ‘right’ answers to the tasks (see the discussion above on meta-reasoning), and without giving them explicit guidance about normative standards we leave them to attain their own reflective equilibrium. Experimenters generally instruct participants what they *should* do when engaging in the task. For example, Cherubini et al. (1998) instructed participants by noting that: “Conclusions should follow from the statements only, and should be certain direct consequences of them … You should therefore try to ignore any knowledge of what the premises are about and try to reason as if they were true” (p. 186). If experimenters direct participants to engage with a task in particular ways then there is often an explicit ‘ought’ as to the answers they are asked to provide. Moreover, it is generally indicated that there are correct solutions, as can be seen in the following instructions from a study by Morley et al. (2004): “This experiment is designed to find out how people *solve* logical problems … Please take your time and be sure that you have the *logically correct answer* before deciding” (p. 8, italics added for emphasis). Even the most recent reasoning papers continue to use phrases such as, “If you judge that the conclusion necessarily follows from the premises, you should answer ‘Valid,’ otherwise you should answer ‘Invalid’ …” (Trippas et al., 2014, p. 11). We suggest that without instructing participants that there is a correct or valid answer to a given problem it is unlikely that standard effects from the reasoning literature would arise. More generally, we contend that it is actually very difficult to envision a way in which to present ‘value-free’ instructions in any meaningful sense.

When presented with reasoning instructions – whether these involve explicit directives or implicit hints that there is a ‘correct’ solution – participants may not generate answers that conform to intended normative benchmarks, but instead may provide personally justifiable responses, based upon their understandings of the task. Some participants take longer than others over the given tasks, suggesting they have set more stringent personal thresholds of reasoning adequacy. Others may find that their intuitive responses are satisfactory (e.g., to dismiss what is unbelievable or to endorse what is intuitive). Indeed, for some there appears to be little reasoning analysis taking place at all, as arises with the fastest responders who often seem to lack any disposition to engage in reflective, analytic, Type_2_ thinking when confronted with reasoning tasks. In examining such individual variation we again argue that the best way to inform and enrich theoretical proposals is by triangulating a multiplicity of measures (e.g., response times, confidence judgments, and thinking dispositions) in a way that is informed by normative benchmarks (see above; cf. Ball, 2013a). Such an approach can be highly informative, provided researchers are cautious regarding disputes over such benchmarks and the dangers of directly equating normative responses with the deployment of analytic processes. The question of arbitrating between competing benchmarks is also considered by Crupi and Girotto (2014), who argue that this lies in the realm of philosophy rather than psychology and that the issue of arbitrating between competing normative standards has not played a particularly significant role in the reasoning literature. We have some sympathy with this observation, but would add that it nevertheless remains interesting and important to investigate the psychological basis for why different reasoners align with different normative standards, as in the case of Wason’s (1966) selection task, where some participants appear to reason according to Oaksford and Chater’s (1994) ‘information gain’ benchmark whilst others appear to reason according to the benchmark of propositional calculus.

On the individual differences theme we also note that since the most academically gifted tend to be those who are more cognitively able, more motivated to find the ‘right’ answer, and less inconvenienced by the need to engage effortful, reflective processing, then it is likely that their responses will correspond with those predicted by normative theories. This is particularly likely to be the case when those responses require additional cognitive effort and motivation, such as occasions where Type_1_ and Type_2_ processes come into conflict and the reasoner concords with a Type_2_ response. The fact that the answers of these reasoners correspond with those of gifted professors of logic and probability who construct normative theories in the first place is, perhaps, unsurprising. Where such evidence converges, we suggest that it is warranted to make claims about whether answers arose through intuitive or analytic thinking, especially if such answers are associated with increased response times. Results of this kind are not always so neat, but we suggest they do warrant theory development and the generation of hypotheses. Moreover, they are also given valuable context by the existence of normative theories. Indeed, in a case where an individual responds with a logically necessary conclusion to a multiple-model syllogism^6^, which is produced after an extended period of deliberation, through the consideration of alternative representations and the application of considerable cognitive effort, it would seem unreasonable to judge it as being of equal value to a response produced intuitively, and rapidly that may have involved very little reasoning. From a Meliorist perspective, it is clear that this effortful consideration of multiple models is more desirable than an intuitive, non-logical response and such results provide both an interesting context for normative theories and evidence of further sub-sets of behavior that a descriptivist must account for in their theorizing.

We contend that it is psychologically interesting to investigate reasoners who understand and engage with the experimenter’s instructions, reasoners who adopt more nuanced interpretations of quantifiers, and reasoners who actively consider alternative representations and counterexamples – particularly those who do this without formal instruction in the relevant normative theory. We would argue that an abandonment of the research program into the psychological correlates of normative reasoning would be a far more damaging than the potential for theoretical cul-de-sacs that can be generated when philosophical and psychological questions are conflated.

## NORMATIVISM AS A SUB-CATEGORY OF INSTRUMENTAL RATIONALITY

An appeal to soft normativism seems to be reflected in Elqayam’s (2012) more recent development of a metatheoretical framework that she describes as *grounded rationality,* which involves an extension of her earlier purely descriptivist position (e.g., Elqayam and Evans, 2011). Elqayam’s (2012) grounded rationality proposal involves her acceptance of a ‘moderate epistemic relativism,’ that is, the view that any description of behavior or cognition as rational needs to be grounded by the context in which it takes place. Thus, for example, a slow analytic judgment will always be irrational if it is made too late to be relevant. We agree with Elqayam’s (2012) position regarding moderate epistemic relativism, but we take issue with a key aspect of her grounded rationality account, which only allows for a very narrow role for normativism in judging behavior or cognition. The argument is that in order for an inference to be considered as normative the reasoner must adopt the goal of reasoning in accordance with a particular normative theory, with the adoption of such a goal presumably being a *conscious* process. In this way “normative rationality can still be evaluated, albeit as a sub-category of instrumental rationality” (Elqayam, 2012, p. 628). The explicit adoption of a normative theory as an epistemic goal by a reasoner would seem to be an exceptionally rare circumstance. It is far more likely that someone consciously sets out to reason or argue ‘rationally’ or ‘correctly,’ but that their knowledge or application of a particular set of normative standards is merely implicit to this goal. Indeed, untrained participants often demonstrate deductive competence when their responses are judged according to logical principles, but this does not mean that their goal was to respond in accordance with a normative system such as logic, nor does it mean that explicit knowledge of logical principles was applied in the production of normatively consistent responses.

Given Elqayam’s (2012) apparent proposal that explicit awareness of a normative benchmark is necessary for a reasoning process to be designated as normative – either from a grounded rationality perspective (Elqayam, 2012) or from a Rationality_2_ perspective (Evans and Over, 1996) – then the attainment of such normativity by a reasoner would only be available to elite participants who have been trained in, for example, formal logic or Bayesian probability. The untutored will be unlikely to recognize explicitly their analytic thinking as conforming to these criteria and so cannot be described as conforming to Rational_2_ standards. In fact, we would only really be able to claim that someone has been Rational_2_ if we asked them after an experiment to tell us which normative standard they were following and they were able to describe this normative standard successfully. Therefore, untutored participants who, during an experiment, set their instrumental goal to follow the instructions, to consider carefully every state of affairs that they can bring to mind and to respond rationally, cannot be considered Rational_2_ according to Elqayam’s proposal. Instead, they would be classified as having produced normative responses via Rational_1_ processes. The result is, we contend, an incredibly narrow conception of Rationality_2_, whereby it virtually never occurs in standard reasoning research, where participants are almost always selected because they are naïve to formal logic or some other normative benchmark.

Evans (2007) makes it clear that analytic Type_2_ thinking is not synonymous with Rational_2_ thinking. This is not simply because analytic thinking does not always align with normative responding, but because Type_2_ thinking does not necessarily (or even often) include the conscious goal to reason in accordance with a specific set of normative benchmarks. Moreover, we argue that it should not be claimed that someone is Irrational_2_ due to their ignorance of normative benchmarks. If someone is responding in the absence of a normative benchmark, rather than contravening a standard that they are aware of, they may be better conceived of as Arational_2_; only someone who is aware of the appropriate normative theory, but who then fails in their application of it, can be considered to be Irrational_2_. Participants who avoid the fundamental analytic bias, but are not trained in a particular normative theory are, we argue, very valuable to the development of reasoning theory and are central to the Meliorist agenda. They do not, however, fit neatly into either category of rationality.

While claims that thinking reflects some normative system or that thinking ought to conform to a normative system remain controversial, we argue that thinking can be usefully contrasted with relevant normative systems and that such comparisons inspire and advance the study of the psychology of reasoning. These comparisons should be made with an assumption of bounded rationality (Simon, 1982), that is, with due consideration to the computational demands of tasks and the pragmatic interpretations that people adopt, as well as a realistic stance on the cognitive capacities that we possess. As Stich (1990) famously argued “it seems simply perverse to judge that subjects are doing a bad job of reasoning because they are not using a strategy that requires a brain the size of a blimp” (p. 27). Evans and Elqayam (2011) acknowledge that “paradigms inspired by normativism have led to a number of important psychological findings” (p. 283), and we concur that while these normative theories do not provide perfect foundations for psychological theories of reasoning to be built upon, they do remain a useful benchmark against which to consider participants’ reasoning. Furthermore, scrutiny of Meliorist theories from a descriptivist perspective as well as scrutiny of descriptivist theories from a Meliorist perspective has the potential to offer insights for enhancing reasoning and for furthering our ability to describe and understand the cognitive processes that reasoning is underpinned by. In sum, we accept that there are numerous issues with taking an uncritical approach to the use of normative standards in reasoning research, but we also argue that if such standards are discarded altogether we lose the proverbial baby with the bathwater, which undermines our explanations and descriptions of reasoning processes to the point of potential triviality.

## Conflict of Interest Statement

The authors declare that the research was conducted in the absence of any commercial or financial relationships that could be construed as a potential conflict of interest.

## Footnotes

1 It might be argued that those tasks that do not correlate with other tasks are examples of ones that give rise to ‘cognitive illusions,’ as described by Cohen (1981), or else are tasks where specific ‘mindware’ (i.e., specialized cognitive rules or strategies; see Stanovich, 2009) is more important than more general reasoning ability.

2 This is an example of an issue of scalar implicature, as discussed by Grice (1975), whereby there is a clash between the quality of the information provided and then quantity of information provided. In a cooperative social exchange the use of the quantifier ‘Some’ when it is possible to use the quantifier ‘All’ violates Gricean maxims of effective communication.

3 Belief-bias is a pervasive tendency in reasoning to accept believable conclusions more frequently than conclusions that contradict beliefs, irrespective of the logical validity of conclusions (see Evans et al., 1983, for pioneering research on this phenomenon that also established the standard ‘belief-bias paradigm’ that inspired most subsequent research).

4 Another interesting methodology that is being used increasingly in the study of reasoning concerns the measurement and analysis of autonomic arousal (e.g., De Neys et al., 2010; Morsanyi and Handley, 2012), which appears to reveal participants’ implicit awareness of reasoning conflicts (e.g., between the logical status and belief status of conclusions).

5 The concept of ‘instrumental goals’ relates to Evans and Over’s (1996) notion of Rationality_1_, that is, ‘instrumental’ or ‘pragmatic’ rationality, defined in terms of thinking or deciding in a way that is generally reliable and efficient for achieving one’s personal goals. As such, Rationality_1_ extends to genetically hard-wired procedures and experientially acquired processes that are automatic and implicit in nature. Evans and Over (1996) contrast the concept of Rationality_1_ with Rationality_2,_ with this latter type of rationality being defined in terms of acting when one has a reason for what one does that is sanctioned by a normative theory. This means that the individual is not merely complying with normative rules in an implicit manner, but is following such rules explicitly.

6 A multiple-model syllogism is a cognitively demanding reasoning problem where multiple possibilities need to be considered to be certain of what necessarily follows according to formal logic (e.g., see Johnson-Laird and Byrne, 1991).

## GLOSSARY

### ARATIONAL

Neither rational nor irrational, but instead existing outside of the category of rationality.

### BELIEF BIAS

The tendency to judge the validity of an argument based on the believability of its conclusion rather than on whether the conclusion is logically necessitated by the argument’s premises.

### DESCRIPTIVISM

The view that normative standards are not appropriate benchmarks in cognitive science and that the goal of psychological research is to *describe* behavior without making value judgments.

### DOUBLE NEGATION ELIMINATION

The inference that if not not-A is true then A is true (and its converse), which is proposed by Rips (1994) as a simple, intuitive logical rule.

### MELIORISM

In general usage, Meliorism is the belief that humans can improve the world. In the present context the term is used specifically to refer to the idea that thinking, reasoning and judgment can be enhanced through education, training and practice. Meliorism in this latter sense also reflects a research program in Cognitive Science.

### NORMATIVE

Refers to the ‘correct’ answer or the ‘right’ way of doing things. In the present context, normative benchmarks are the (often debatable) standards for thinking, reasoning or deciding that participant responses tend to be evaluated against. These normative benchmarks derive from formal, logical systems or probability theory.

### PANGLOSSIAN

Derived from Dr. Pangloss, the eternal optimist in Voltaire’s *Candide*, Panglossian refers to the belief that ‘all is for the best in the best of all possible worlds.’ In the present context, it is the idea that we have the best of all possible cognitive systems.

### RATIONALITY_1_

Thinking, speaking, reasoning, making a decision, or acting in a way that is generally reliable and efficient for achieving one’s goals.

### RATIONALITY_2_

Thinking, speaking, reasoning, making a decision, or acting when one has a reason for what one does sanctioned by a normative theory.

### SLIPPERY SLOPE FALLACY

The argument that a relatively small first step leads inevitably to the bottom of the slippery slope, so if A happens then B will happen and if B happens then C will happen, all the way down to the terrible scenario of Z.
